# What the Erythrocytic Nuclear Alteration Frequencies Could Tell Us about Genotoxicity and Macrophage Iron Storage?

**DOI:** 10.1371/journal.pone.0143029

**Published:** 2015-11-30

**Authors:** Juliana M. M. Gomes, Heder J. Ribeiro, Marcela S. Procópio, Betânia M. Alvarenga, Antônio C. S. Castro, Walderez O. Dutra, José B. B. da Silva, José D. Corrêa Junior

**Affiliations:** 1 Instituto de Ciências Biológicas, Departamento de Morfologia, Universidade Federal de Minas Gerais, 31270–901, Belo Horizonte, MG, Brazil; 2 Faculdade de Medicina, Departamento de Medicina, Universidade Federal de Juiz de Fora, 35010–177, Governador Valadares, MG, Brazil; 3 Faculdade de Medicina, Departamento de Medicina Veterinária, Universidade Federal de Juiz de Fora, 36036–900, Juiz de Fora, MG, Brazil; 4 Instituto de Ciências Exatas, Departamento de Química, Universidade Federal de Minas Gerais, 31270–901, Belo Horizonte, MG, Brazil; Federal University of Rio de Janeiro, BRAZIL

## Abstract

Erythrocytic nuclear alterations have been considered as an indicative of organism’s exposure to genotoxic agents. Due to their close relationship among their frequencies and DNA damages, they are considered excellent markers of exposure in eukaryotes. However, poor data has been found in literature concerning their genesis, differential occurrence and their life span. In this study, we use markers of cell viability; genotoxicity and cellular turn over in order to shed light to these events. Tilapia and their blood were exposed to cadmium in acute exposure and *in vitro* assays. They were analyzed using flow cytometry for oxidative stress and membrane disruption, optical microscopy for erythrocytic nuclear alteration, graphite furnace atomic absorption spectrometry for cadmium content in aquaria water, blood and cytochemical and analytical electron microscopy techniques for the hemocateretic aspects. The results showed a close relationship among the total nuclear alterations and cadmium content in the total blood and melanomacrophage centres area, mismatching reactive oxygen species and membrane damages. Moreover, nuclear alterations frequencies (vacuolated, condensed and blebbed) showed to be associated to cadmium exposure whereas others (lobed and bud) were associated to depuration period. Decrease on nuclear alterations frequencies was also associated with hemosiderin increase inside spleen and head kidney macrophages mainly during depurative processes. These data disclosure in temporal fashion the main processes that drive the nuclear alterations frequencies and their relationship with some cellular and systemic biomarkers.

## Introduction

Erythrocytic nuclear alterations are excellent markers of genetic instability due to their advantages such as simplicity, reliability, and sensitivity [1–4]. Despite these characteristics, it is known that their frequencies in blood depend on two main factors associated to their genesis and their removal from the blood stream. In fish, their genesis occur in head kidney portion in which is located their hematopoietic tissue while the erythrocyte removals is associated to different organs such as liver and spleen depending on the species [5–9].

The low nuclear alterations frequencies achieved by organisms exposed display a specific control on their occurrence that mismatch many others markers of cellular viability. Whilst the relative frequencies could be associated to specific genotoxic inducers and species, no systematic studies dealing with their dynamic has performed to dates [3, 10, 11].

The aquatic environment has been contaminated by numerous toxic agents that are usually produced by industrial, agricultural, and domestic activities resulting from inappropriate use of water resources. This pollution contributes to the degradation of various environmental compartments (water, sediments, air, and soil) and affects the health of local individuals [12]. Heavy metals such as cadmium are highly dangerous to the ecosystem because of their persistence, bioaccumulation, and genotoxicity, with effects observed even at trace concentrations [13, 14].

The cadmium has been identified to decrease levels of cellular antioxidants [15–17], damaging DNA, proteins, and membranes [18–21]. In the nuclei, damage can occur through DNA adduct formation, DNA/DNA, and DNA/protein cross-links, and DNA single/double strand-breaks, in addition to defects in the repair mechanisms. Chromosomal damage had characterized as result of deficient DNA repair, usually occurring during cell division with an increased extent of genotoxic accumulation. Such damage promotes the inhibition of DNA replication and repair through decreased fidelity of DNA polymerases [3, 22].

Some of these genotoxic effects can be measured through the frequency of nuclear alterations using light microscopy, as observed for fish erythrocytes exposed to pollutants that altered the nuclei [3, 22]. Nuclear alterations (NAs) in nucleated erythrocytes had been described based on their morphological characteristics [23, 24] and nuclei identified as blebbed, bud, condensed, lobed, micronuclei, notched, and vacuolated. The micronucleus (MN) test is the most widely used because it detects the genotoxicity of a wide range of compounds, especially in fish [2, 25, 26]. The use of NAs as indicators of genotoxic damage has not been as extensively studied as micronuclei [10, 14, 27, 28].

Cadmium-induced stress is also associated with peroxidation of lipids. Such damage induces changes in permeability, fluidity, and ion transport; inhibition of metabolic processes; modifications of transmembrane potential (depolarization); release of mitochondrial calcium; and uncoupling and activation of caspase-3, generating fragmentation of DNA that could lead to apoptosis [15, 17, 29, 30]. Intracellular contaminants directly induce these effects through lipid peroxidation, which causes cross-linking and polymerization of membrane components, thus altering membrane functions. The unsaturated fatty acids present in the membranes (phospholipids, glycolipids, and sterols) and transmembrane proteins containing oxidizable amino acids are especially susceptible to damage caused by free radicals [31]. This aspect highlights that membrane integrity is an important barrier for intracellular homeostasis. Structural damage to lipid or protein components of the membrane allows xenobiotics to enter the cell [15, 32, 33]; however membrane integrity over time associated with Cd exposure has been minimally addressed in the literature for aquatic organisms.

Tilapia (*Oreochromis niloticus*) is useful for bioassays because of its high prolificacy, resistance to many diseases rapid growth, and hardiness [34]. As an environmental bioindicator, tilapia present easy adaptation to new environments, responds well to chemicals, has a widespread distribution and is used as protein resource for humans [35, 36].

Although some studies have shown membrane permeability and ROS as results of toxicity, the permeability is not associated to the different categories of erythrocytes nuclear alteration (ENAs). Therefore, the objective of this study was to investigate the ENAs behavior facing the genotoxic effects induced by Cd exposure and their systemic influence concerning turn over, iron retention and whole blood cadmium content. We intend to shed light on changes in the frequency of the different types of ENAs, including those during a depurative process.

## Materials and Methods

### Biological assays

The fish were acquired from LAQUA (Laboratório de Aquacultura da Escola de Veterinária-UFMG), transported in plastic bags with aerated tap water. The animals were acclimated under laboratory conditions (ICB-UFMG) for 3 weeks in 40 L plastic aquaria (polypropylene, polystyrene, and bisphenol A-free) with dechlorinated water (22°C) and a 12/12 h light/dark photoperiod. The aquaria were previously washed with 10% nitric acid and rinsed three times with distilled water. Before the procedures the fish were anesthetized with 25 mg L^-1^ of Eugenol (Biodinâmica Europa S.L.^®^) dissolved in tap water [37]. The animals were carefully handled and maintained in hydrated sponge before each subsequent procedure. Less than 30 seconds were spent in caudal venipuncture procedures [28, 38] for Erythrocytes lysis, Ficoll gradients, *Ex vivo* assay and *In vivo* assay and euthanasia necessary for the tissues sampling. All procedures were performed according to the animal health care guidelines submitted and approved by the Ethics Committee on Animal Experimentation (CETEA—UFMG) under protocol number 240/2010.

### Erythrocyte-rich population (ERP) determination in peripheral blood by flow cytometry

#### Erythrocytes Lysis

Peripheral blood (0.5 mL) was obtained from *O*. *niloticus* using a syringe with 0.1 mL of solution containing 0.25 M EDTA (ethylenediamine tetraacetic acid) at pH 6.8 [39]. Blood samples were diluted in a 3x volume of red blood cell (RBC) Lysis Buffer (Sigma Aldrich). This buffer was prepared by diluting one portion into nine parts of water before being mixed with the blood. The blood sample was incubated in RBC buffer for 15 minutes at room temperature. The samples were resuspended in 10 ml of phosphate-buffered saline (PBS—water-based salt solution containing 0.26g of NaH_2_PO_4_.H_2_O_,_ 2.17g of Na_2_HPO_4_.7H_2_, 11,69g of NaCl in 1 liter of deionized water) and washed by centrifugation (400 g, 4°C, for 10 minutes). Measurements were performed using a flow cytometer (Becton Dickinson FACScan), producing graphs for FSC (forward-scattered) x SSC (side-scattered) [40]. A total of 150,000 cells was analyzed.

#### Ficoll

Peripheral blood (2 mL) was collected with a 4:1 ratio (blood/EDTA) for obtaining different blood populations. The whole blood volume collected was placed in a mixture of Ficoll–Hypaque (Sigma Chemical Co., USA, density: 1.119 g/mL) and Ficoll–Hypaque (Sigma Chemical Co., USA, density: 1.077 g/mL) at a 1:3 ratio (Ficoll/blood) in sterile polystyrene conical bottom tubes (Falcon™, Corning®, USA). All samples were centrifuged at 400 g for 40 min at 22°C. The ring of lymphocytes and thrombocytes was collected and acquired in flow cytometer in order to confirm their location. The same process was performed for the erythrocytes and granulocytes rich portion [41].

#### Assessment of ROS and membrane disruption of ERP *ex vivo* assay

Cells were obtained from peripheral blood, collected with syringes with a 4:1 ratio blood/heparin solution (Hepamax-s—Heparin 5000 IU mL). The cell cultures were performed on culture plates (24 wells) containing 1 mL culture medium (Dulbecco's Modified Eagle's Medium–Sigma Aldrich plus 1% of penicillin/streptomycin—Gibco). The experiments were conducted in a laminar flow hood (VECO) and the cells were incubated in a CO_2_ incubator (28°C). In each well / treatment, 10 μL of peripheral blood was inserted on culture medium. We used 6 wells / treatment divided into two exposure times (24 and 48 hours) and two cadmium concentrations (2 and 0.2 μL L^-1^) plus respective controls [42].

After each time the cultures were collected, marked by DHE (dehydroethidium—5μM) to assess reactive oxygen species (ROS) and PI (propidium iodide 5μM) to quantify the frequencies of cells with membrane disruption. The cells were analyzed by flow cytometry (FACScan—Becton Dickinson) using an air-cooled 488 nm argon laser, the maximum fluorescence excitation and emission is for PI 535 and 617 nm and DHE of 605 and 518 nm. Data were analyzed using the Software® FlowJo [42, 43].

### *In vivo* assays: tilapia exposure

A total of 63 specimens of *O*. *niloticus* was randomly assigned to one of 9 treatments with 7 animals *per* group. Prior to the experiments, the fish were acclimated under the described laboratory conditions. A semi-static system with forced aeration was used in all treatments. During the assays, 50% of the total water volume was renewed every 48 hours and the cadmium levels were corrected by adding the appropriate Cd stock solution in order to maintain the cadmium concentration in each treatment. The physical-chemical characteristics of water (temperature, oxygen, pH, and ammonia) were measured daily. In order to avoid hormonal, sexual or metabolic influence on our results, the animals used were at juvenile stage with characteristic length of animals that have not reached the stage of sexual maturity [44].

The animals were exposed to two different concentrations of cadmium: 0.25 and 2.5 mg·L^-1^ (Cd-0.25 and Cd-2.5), plus the control (no cadmium). These treatments were evaluated at 48 h and 96 h of exposure (48E and 96E), and exposure for 96 h was followed by 48 h in tanks containing clean water (96E+48D). This latter group (96E+48D) was considered as the depuration time group. At each experimental time point (48E, 96E and 96E+48D), all animals from each group were anesthetized with 25 mg L^-1^ of Eugenol (Biodinâmica Europa S.L.^®^), weighed, and measured [37]. Peripheral blood samples were obtained from the caudal vein of the specimens as described above [28, 38; 39]. Animals were killed by cervical scission [45], and no food was supplied to the fish during the experiment in other to prevent their influence on results [46; 47].

#### Assessment of membrane disruption of ERP in *in vivo* assay

Blood cells with compromised membrane integrity were detected by a flow cytometric assay using propidium iodide staining. Briefly, blood cell in 25 μl aliquots were incubated for 15 minutes with 3 μl of propidium iodide (50μg/ml) (PI—Sigma) in the dark. The samples were resuspended in 1 ml of PBS and immediately analyzed (without fixation) by flow cytometry using a BD FACScan flow cytometer (Becton Dickinson) as described above. The propidium iodide fluorescence of individual nuclei was examined in 30,000 cells *per* animal. All FACS parameters (FSC and SSC) and region settings were kept identical throughout all experiments. The data were analyzed using FlowJo software^®^ [42].

#### Erythrocyte nuclear alteration (ENA) analysis in *in vivo* assay

Smears were prepared on air-dried slides and stained with May-Grunwald for 6 min. Four slides were prepared for each fish and 2,000 cells *per* fish were counted under a microscope using an immersion objective (100x) [48], for a total of about 14,000 cells *per* treatment. Small, non-refractile, circular or ovoid chromatin bodies showing the same staining pattern as the main nucleus and clearly separated from the main nuclei were considered as micronuclei (MN). ENAs were classified according to [23]. Briefly, nuclei displaying a relatively small evagination of the nuclear membrane along with the euchromatin were considered as blebbed (BL). Evaginations larger than the blebs were classified as lobed (LO). Nuclei with vacuoles were recorded as vacuolated (VA). Nuclei showing a deep invagination toward the center were considered as notched (NO). Nuclei with condensed chromatin were classified as condensed (CO), and those having a small bud were considered as bud (BU). Only cells clearly single from the surrounding cells were scored.

#### Water and blood sampling by atomic absorption spectrometry

Water samples were collected from all the aquarium tanks. The collection containers were polyethylene tubes previously decontaminated in 10% nitric acid solution for 48 h. Water samples were kept in 5% nitric acid in a refrigerator at 10°C until further analysis. Three replicates were performed for each treatment. The calibration points for water were 1.25; 1.5; 2; 2.5; 5; 10; 15; 20; 25 μg L^-1^. LD = 0.1 μg L^-1^ and LQ = 0.3 μg L^-1^.Blood samples were collected from all fish in all treatments. For blood samples, the calibration points were 1.25; 1.5; 2; 2.5; 5; 10; 15; 20; 25 μg L^-1^. LD = 0.6 μg L^-1^ and LQ = 2.0 μg L^-1^.Five replicates were performed for each treatment. The blood was conditioned in decontaminated polyethylene tubes and kept in a refrigerator at 10°C until used for GF-AAS.

The integrated absorbances were determined in an atomic absorption spectrometer, SpectrAA 240 from Varian (Victoria, Australia), equipped with a graphite furnace with integrated platforms (Varian, Part Number 01-900327-0) and a polarized Zeeman background correction. A hollow Cd cathode lamp from Photron (Part Number p808) was operated at 10 mA with a slit of 0.5 nm and a wavelength of 228.8 nm. Argon 99.996% (White Martins, Belo Horizonte, MG, Brazil) was used as the purge gas with a flow rate of 250 mL·min^-1^ [49].

#### Light microscopy and morphometrical analysis in spleen and head kidney

Briefly, tilapias were anesthetized and killed as described above before tissue collection. Pieces organs were fixed in 10% formaldehyde (spleen) and Davidson fluid (head kidney). Tissue fragments were processed for routine paraffin embedding, sectioned at a thickness of 5 μm, deparaffinized with xylene, hydrated in solutions of ethanol and stained by Pearls’ histochemical technique [7]. This enabled the identification of granules containing melanin, hemosiderin, and lipofuscin pigments. With this technique, melanin remains black; hemosiderin stains in blue, and lipofuscin stains yellow-brown [50]. Three slides *per* animal containing spleen and head kidney sections were stained by Pearls’ technique. One section *per* animal was observed using Olympus BX41 microscope with a 40 x objective reticulated eyepiece. The quantification was performed in 20 fields (7220 intersection points *per* fish). The intersection points considered were hemosiderin positive (blue pigment) and for the melanomacrophage centres (MMCs), the total number of intersection point over the centres.

#### Scanning electron microscopy and analytical microscopy (X-ray microanalysis)

For scanning electron microscopy (SEM), sections of spleen and head kidney were mounted on plastic coverslips (Thermanox), metalized with carbon in an evaporator (Hitachi Model HUS4G) and analyzed by SEM with secondary electrons, backscattered electron imaging and X-ray microanalysis in a Quanta FEG 3D (FEI) electron microscope [7].

### Statistical analysis

The data were tested for normality using the Kolmogorov–Smirnov test. The results obtained were analyzed using GraphPad Prism Version 5.0. (San Diego, CA, USA). Results were considered significant when p<0.05. Cadmium levels and macrophages hemosiderin positive were evaluated by ANOVA followed by Bonferroni´s test and expressed as the mean plus the standard deviation. The frequencies of PI-positive cells determined by flow cytometry were evaluated by the Mann-Whitney test in *in vivo* assay and Qui-square test in *ex vivo* assay. The values for PI-positive cells were transformed to percentages to eliminate false-positive results. For ENAs and MMCs, a nonparametric chi-square test was used. The correlation between ENAs and PI-positive cells was determined by Spearman's correlation test.

## Results

### *Ex vivo* assays

#### Determination of ERP in peripheral blood by flow cytometry

**Fig 1:**
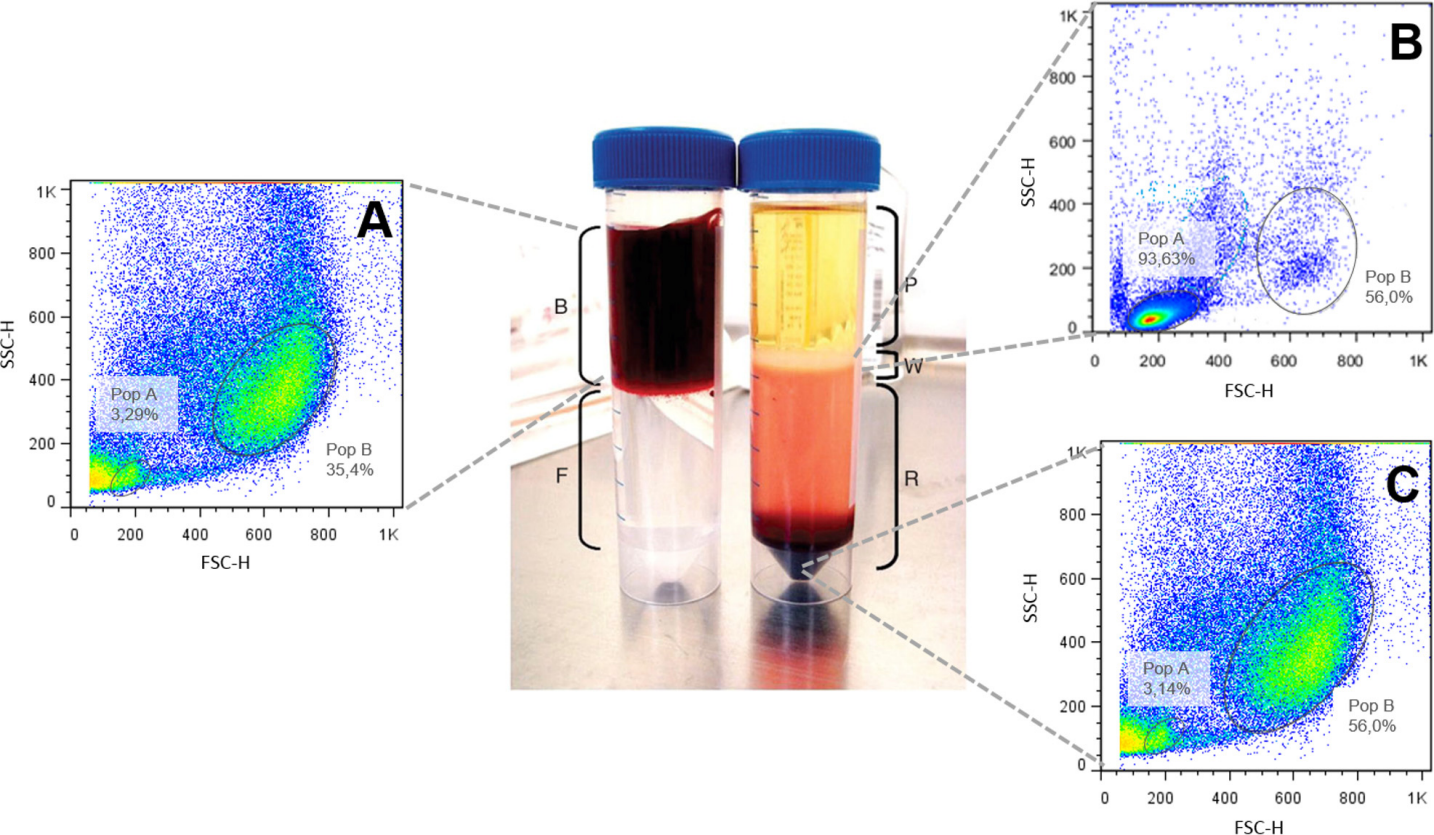
Blood cells populations of *O*. *niloticus* after separation by Ficoll gradient. (A) Whole blood, (B) ring of lymphocytes/thrombocytes—Pop. A and (C) erythrocytes rich population–Pop. B using SSC (side scatter) X FSC (forward scatter) parameters.

Fig 1A shows typical populations distribution and their relative frequencies in the whole blood. After the Ficoll isolation procedures, a characteristic increase in white cells population is observed in the ring of lymphocytes / thrombocytes in Pop. A (Fig 1B). A marked increase in precipitated red cells, richer at the most dense region in Pop B (Fig 1C) is also observed.

**Fig 2:**
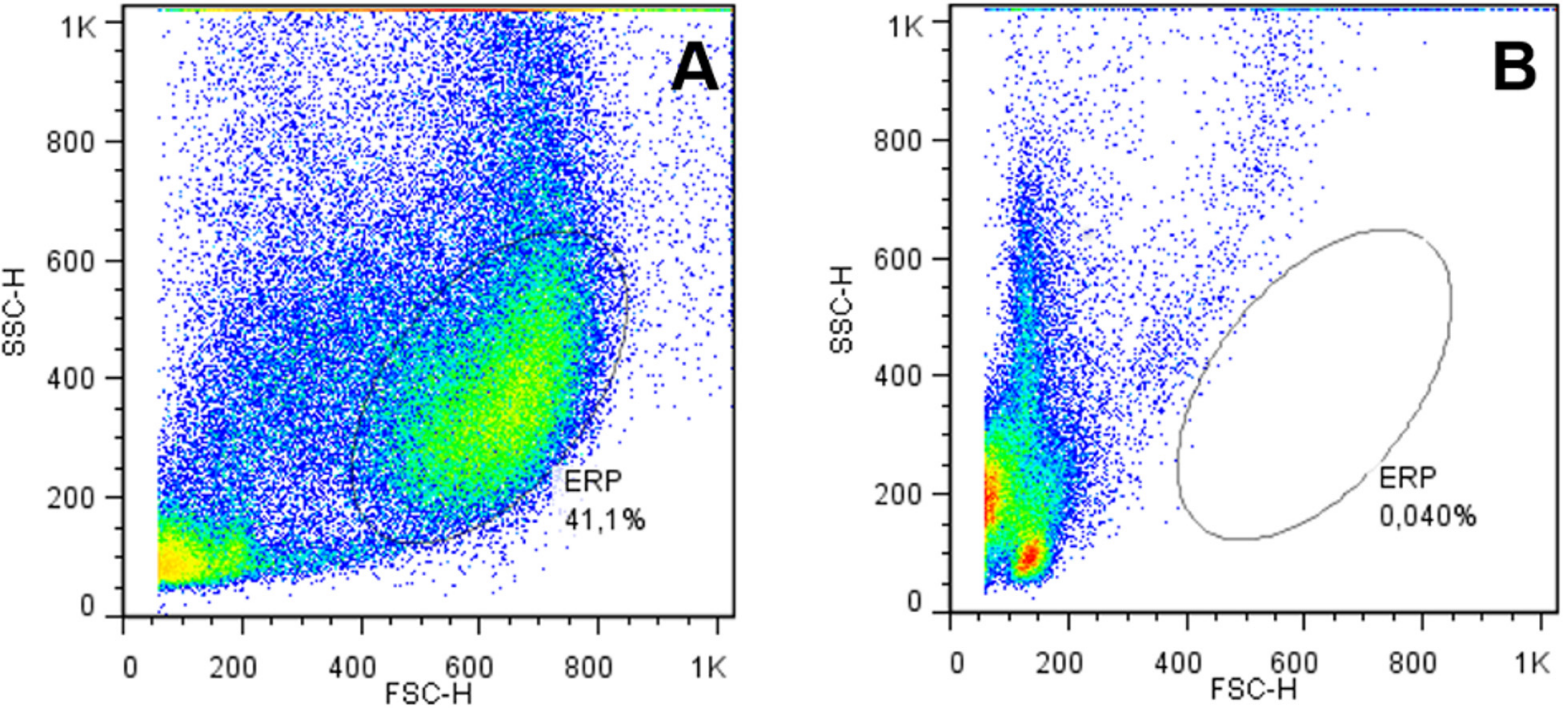
Erythrocytes-rich population (ERP) of *O*. *niloticus*. (A) Normal blood and (B) after erythrocytes lysis using SSC (side scatter) X FSC (forward scatter) parameters.

The erythrocytic population confirmation in cytometric graphs was also estimated by comparing graphs from whole blood cells (Fig 2A) and graphs without erythrocytes (Fig 2B). The ERP disappeared when the erythrocytes were lysed (Fig 2B); thus, this region was confirmed as the erythrocyte-rich population (ERP).

#### Assessment of ROS and membrane disruption of ERP in *ex vivo* assay

To evaluate the reactive oxygen species (ROS), culture of blood cells exposed to different times and concentrations of Cd were marked by DHE and evaluated for mean fluorescence intensity. The exposure concentrations used were correspondent to the values found by spectrometry of atomic absorption for whole blood after *in vivo* assay. MFI of ROS showed increase in the first 24 hours of exposure in both Cd concentrations. However, ROS rates showed basal levels in the subsequent time (Table 1).

**Table 1:** Median fluorescence intensity (MFI) of erythrocytes marked by DHE.

Treatment	Times of exposition	
	24E	48E
**Control**	**22**	**21**
**0.2 μg L** ^**-1**^	**50**	**23**
**2 μg L** ^**-1**^	**46**	**21**

**Fig 3:**
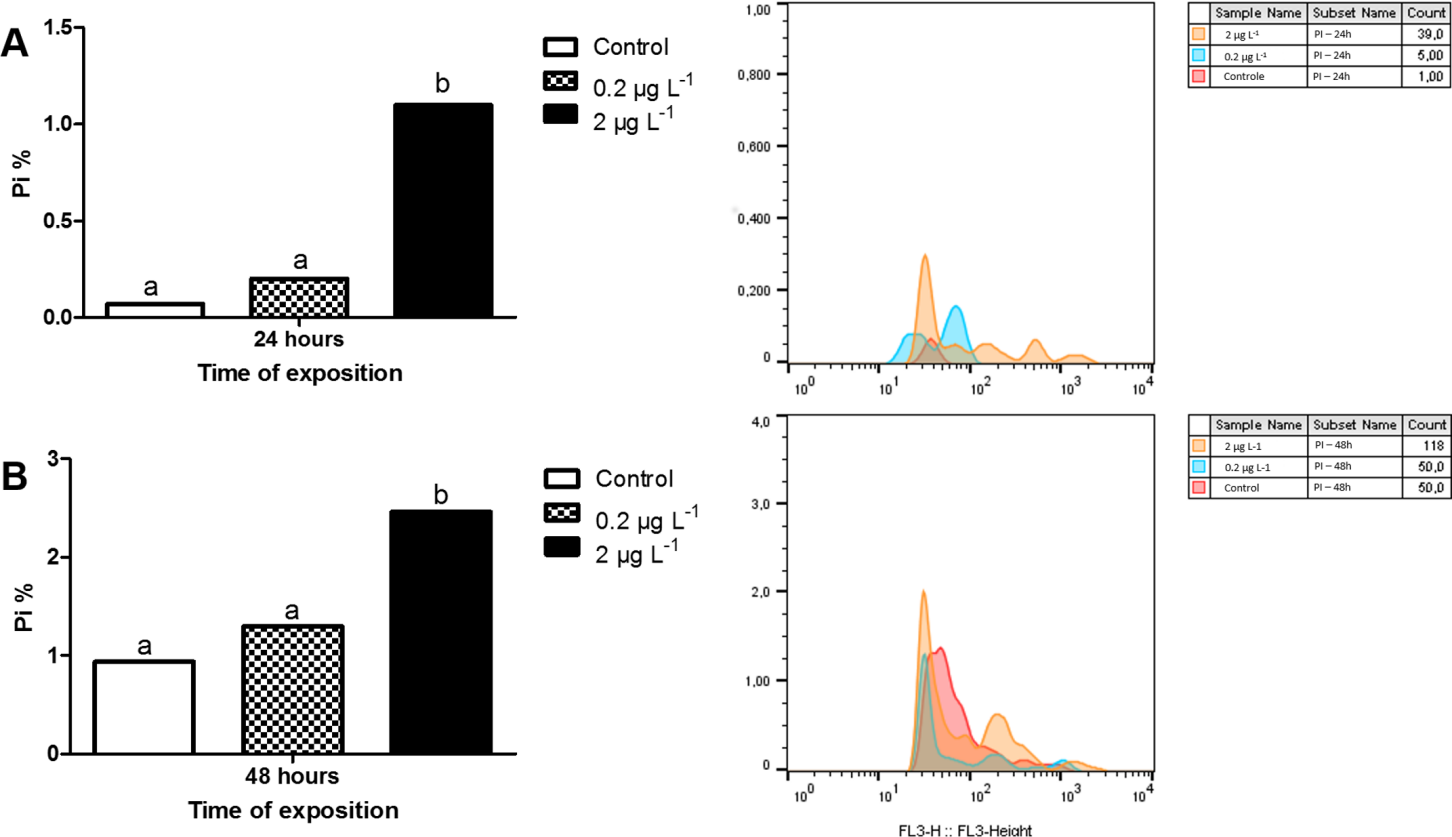
Percentages of erythrocytes labeled by PI in *ex vivo* assay. (A) Exposure to 2 μg L^-1^ and 0.2 μg L^-1^ in 24 and (B) 48 hours. On the right, PI fluorescence histograms 24 and 48 hours respectively. Values followed by different letters differ by Fisher test (p <0.05).

The membrane disruption was detected in ERP by PI stain and analyzed by flow cytometry. In both exposure times, the increase was observed only at the higher concentration (2 μg L^-1^) (Fig 3A and 3B). The histograms (Fig 3A and 3B) showed the PI fluorescence in dose-dependent fashion.

### *In vivo* assays

#### Water physical-chemical parameters

The values for the physical-chemical parameters of water such as temperature, level of dissolved oxygen, ammonia concentration, and pH levels (Table 2) were within the range reported as favorable for the health and survival of animals [44]. The dissolved oxygen concentrations have been at least 95% of the air saturation value throughout the test. Table 3 shows the concentration of cadmium present in each aquarium tank.

**Table 2:** Values (means ± S.D.) of temperature, rate of oxygen, ammonia and pH obtained from the aquaria during the experimental period.

Temperature (°C)	Oxygen (mg^.^L^-1^)	Ammonia (mg^.^L^-1^)	pH
**21.90 ± 0.73**	**7.76 ± 0.25**	**0.71x10^-3^**	**7.72 ± 0.09**

**Table 3:** Expected and determined concentrations (means ± S.D.) of cadmium in water obtained from the aquaria during the experimental period.

		Cadmium determined concentration (μg^.^L^-1^)		
Treatment	Cd expected exposure (μg^.^L^-1^)	48 h	96 h	96E+48D h
**Control**	**0**	**nd**	**nd**	**Nd**
**Cd-0.25**	**250**	**252.00±0.1**	**244.41±0.4**	**8.62±0.3**
**Cd-2.5**	**2500**	**2450.82±4.2**	**2460.10±6.7**	**98.12±1.1**

nd refers to not detectable

#### Animals and biometrics

The animals showed a total length and body weight of 10.05 ± 1.11 cm and 17.36 g ± 5.57, respectively. These values are characteristic of animals that have not reached the stage of sexual maturity [44]. All animals showed a good general appearance, displaying no visible changes on their exterior surface.

#### Analysis of membrane alterations in the ERP in *in vivo* assay

**Fig 4:**
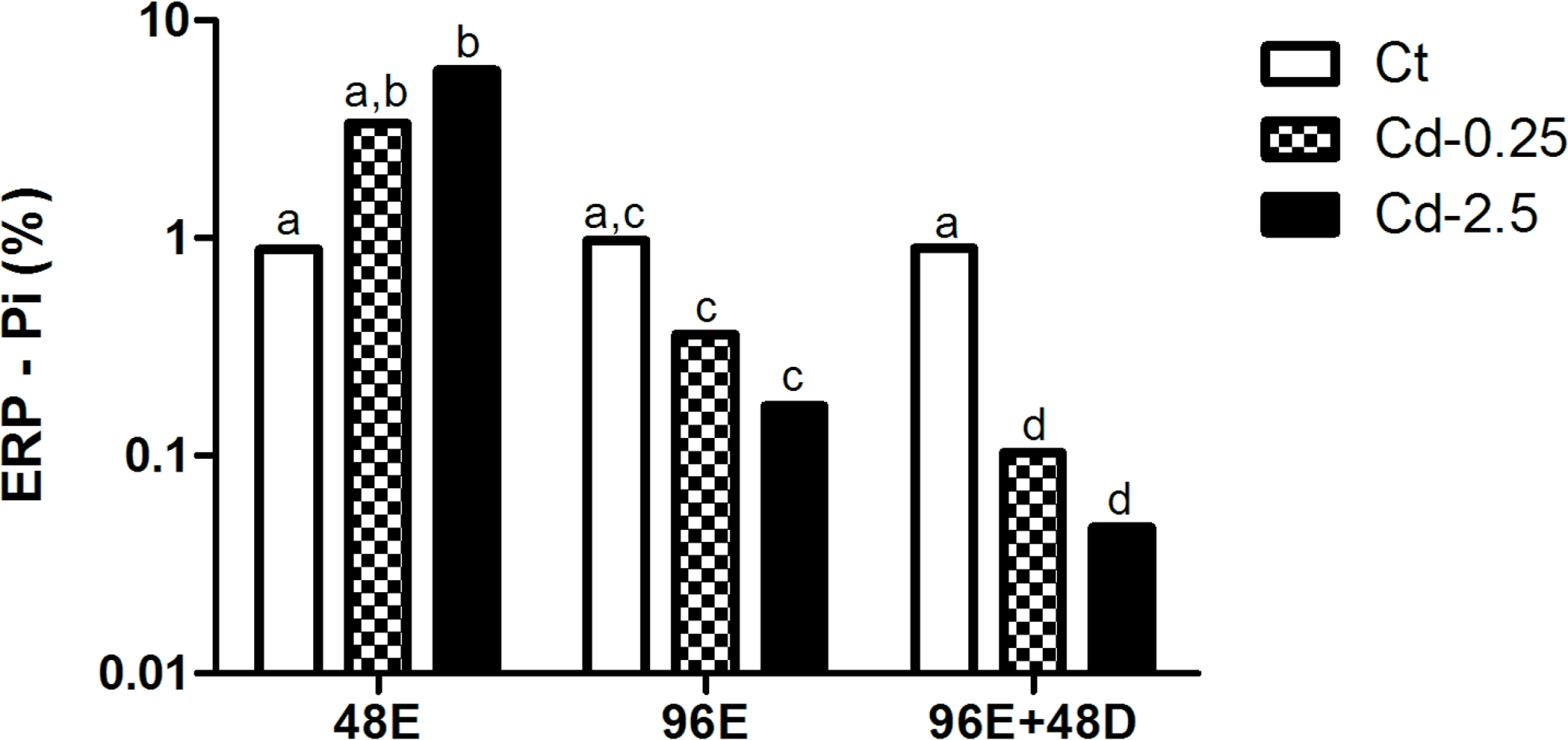
Percentage of erythrocytes labeled by PI in *in vivo* assay. Values followed by different letters differ by Mann-Whitney test (p<0.05).

An increase in the number of cells labeled by PI at 48 hours of exposure and a decrease at the other exposure times was observed at the highest cadmium concentrations (Cd-2.5). Although there was no significant increase at 48 hours at the lower concentration (Cd-0.25), a decrease was observed at subsequent time points (96E and 96E+48D). There was no significant difference between the exposed groups (Cd-0.25 and Cd-2.5) at each time (Fig 4). No difference was noted among controls during the assay.

#### ENAs identifications and relative frequencies

**Fig 5:**
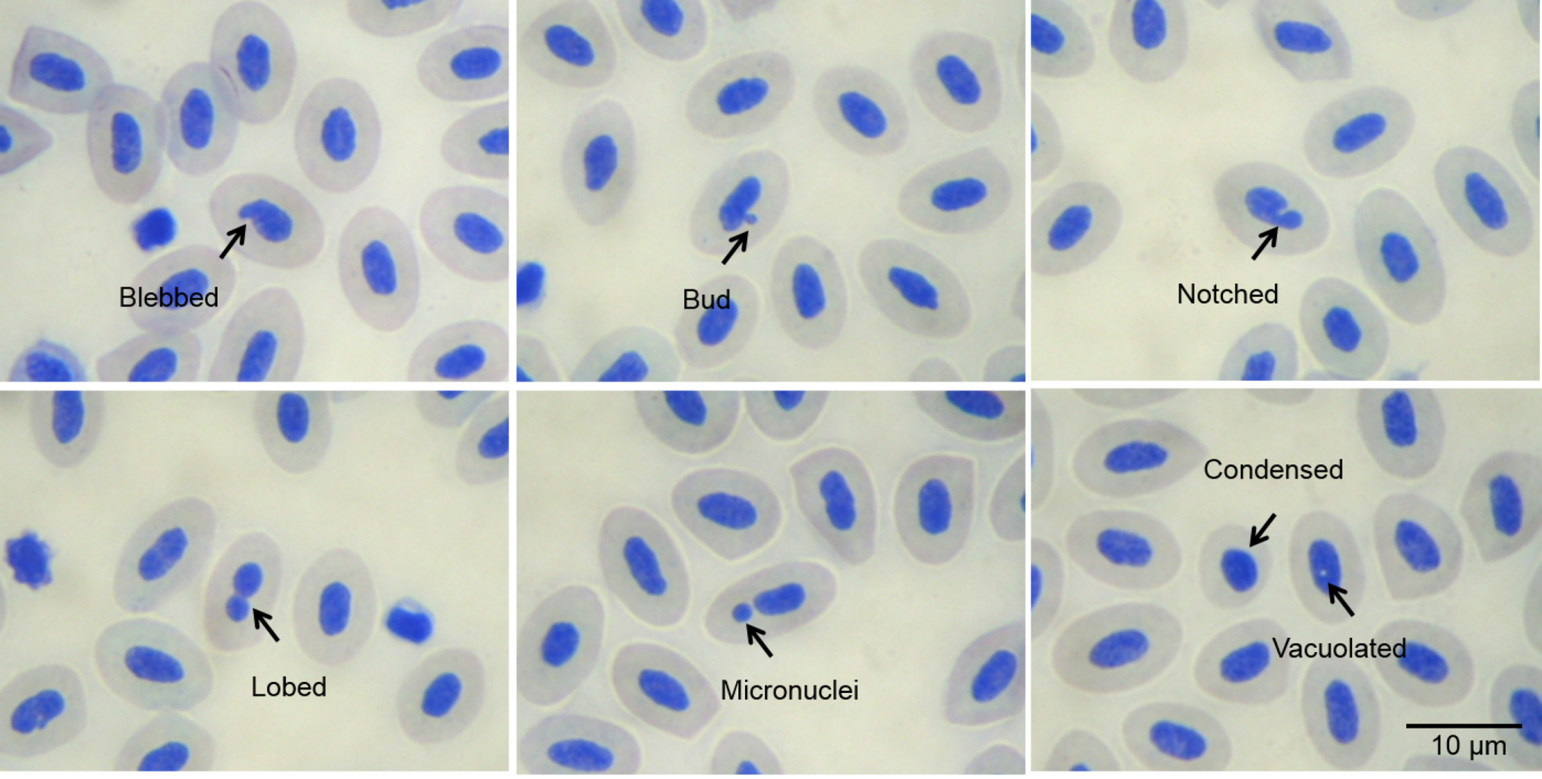
Alterations in erythrocytes of *O*. *niloticus* stained by May-Grunwald in *in vivo* assay.

The erythrocytic nuclei alterations identified in *O*. *niloticus* blood samples were blebbed (BL), bud (BU), condensed (CO), notched (NO), lobed (LO), micronuclei (MN), and vacuolated (VA) (Fig 5).

#### ENAs frequencies

The ENAs lobed, blebbed, and notched showed the highest frequencies in all treatments, followed by bud, vacuolated, condensed, and micronuclei. Micronuclei exhibited the lowest frequency in most of the treatments (Table 4).

**Table 4:** Sequence of occurrence of individual ENAS (%).

	ENAs frequencies (%)		
Treatment	48 hours	96 hours	96E+48D hours
**Control**	**LO>BL>NO>CO>BU = VA>MN**	**LO>BL>NO>BU>MN>VA = CO**	**BL>LO>NO>BU>VA = CO>MN**
	**36>26>18>10>05 = 05>00**	**37>33>19>07>02>01 = 01**	**39>30>21>05>02 = 02>01**
**Cd-0.25**	**LO>BL>NO>CO>BU>VA = MN**	**LO>BL>NO>BU = VA>CO>MN**	**LO>BL>NO>BU = VA>CO>MN**
	**41>25>19>06>05>02 = 02**	**38>34>19>03 = 03>02>01**	**39>31>18>04 = 04>03>01**
**Cd-2.5**	**LO>BL>NO>BU>VA>CO>MN**	**LO>BL>NO>BU = VA>CO>MN**	**BL>LO>NO>BU>VA>CO = MN**
	**35>26>23>09>04>02>01**	**39>31>18>04 = 04>03>01**	**36>31>18>08>05>01 = 01**

LO—lobed; BL—blebbed; NO—notched; CO—condensed; BU—bud; VA—vacuolated and MN—micronuclei. Values below each ENA indicate its frequency. Analysis included 14,000 cells *per* treatment.

#### Correlations among ENAs and PI-positive erythrocytes

In this study, some nuclear alterations were correlated with others and the number of correlations increased with the Cd dose (Table 5). No significant correlations between the ERP marked by PI and nuclear alterations were observed in the control groups and Cd-0.25 group. In the Cd-2.5 group, negative correlations (cells labeled by PI and bud; cells labeled by PI and notched) were observed at 48 hours of exposure and positive correlations (cells labeled by PI and bud; and cells labeled by PI and lobed) were observed during the depuration period (Table 5). Lobed was the only nuclear alteration with a decreased frequency during the depuration period.

**Table 5:** Spearman’s correlation coefficients (R) matrix between nuclear alterations and PI positive ERP.

Sperman’s correlation (R)																					
	48E							96E							96E + 48D						
	MN	BU	BL	LO	NO	VA	CO	MN	BU	BL	LO	NO	VA	CO	MN	BU	BL	LO	NO	VA	CO
**Control**																					
**BU**	**0.674**																				
**BL**	**0.655**	**0.706**																			
**LO**	**0.393**	**0.088**	**0.486**																		
**NO**	**0.133**	**-0.358**	**-0.029**	**0.841**																	
**VA**	**0.270**	**-0.091**	**0.265**	**0.971** **	**0.940** *																
**CO**	**-0.266**	**-0.403**	**-0.058**	**0.377**	**0.544**	**0.448**															
**PI**	**0.354**	**0.369**	**0.000**	**0.500**	**0.462**	**0.564**	**-0.616**														
**Cd-0.25**																					
**BU**	**0.407**							**0.033**													
**BL**	**0.391**	**0.044**						**0.370**	**0.679**												
**LO**	**0.062**	**0.058**	**0.928** *					**0.370**	**0.679**	**1.000** **											
**NO**	**0.814**	**0.441**	**0.662**	**0.464**				**0.376**	**0.626**	**0.986** **	**0.986** **										
**VA**	**0.383**	**-0.313**	**0.344**	**0.123**	**0.016**			**0.063**	**0.204**	**0.754**	**0.754**	**0.721**									
**CO**	**-0.150**	**-0.814**	**0.391**	**0.339**	**0.016**	**0.183**		**0.254**	**0.318**	**0.618**	**0.618**	**0.627**	**0.448**								
**PI**	**-0.289**	**0.359**	**0.462**	**0.700**	**0.051**	**-0.447**	**0.224**	**-0.224**	**-0.791**	**-0.500**	**-0.500**	**-0.564**	**0.051**	**0.105**							
**Cd-2.5**																					
**BU**	**0.853** *							**0.361**							**0.226**						
**BL**	**0.828**	**0.677**						**-0.062**	**-0.516**						**-0.034**	**0.896** *					
**LO**	**0.621**	**0.883** *	**0.657**					**-0.141**	**-0.462**	**0.986** **					**0.135**	**0.971** **	**0.928** *				
**NO**	**0.840**	**0.985** **	**0.754**	**0.928** *				**0.123**	**-0.334**	**0.771**	**0.725**				**0.541**	**0.912** *	**0.696**	**0.829**			
**VA**	**0.880** *	**0.907** *	**0.880** *	**0.880** *	**0.955** *			**0.237**	**-0.359**	**0.845** *	**0.857** *	**0.541**			**0.072**	**0.375**	**0.062**	**0.395**	**0.334**		
**CO**	**0.660**	**0.313**	**0.516**	**-0.030**	**0.308**	**0.419**		**-0.590**	**-0.097**	**-0.152**	**-0.062**	**-0.698**	**-0.072**		**-0.480**	**-0.139**	**-0.051**	**-0.169**	**-0.101**	**-0.287**	
**PI**	**-0.866**	**-0.975** *	**-0.500**	**-0.900**	**-0.975** *	**-0.894**	**-0.410**	**0.527**	**0.205**	**-0.600**	**-0.600**	**-0.800**	**-0.224**	**0.154**	**0.447**	**0.975** *	**0.872**	**1.000** *	**0.800**	**0.447**	**-0.707**

BL—blebbed, BU—bud, CO- nuclear condensed, NO—notched, LO—lobed, MN—micronuclei, VA—nuclear vacuolated and PI—PI-positive cells.

* Indicates (p <0.05) and

** indicates (p<0.005).

**Fig 6:**
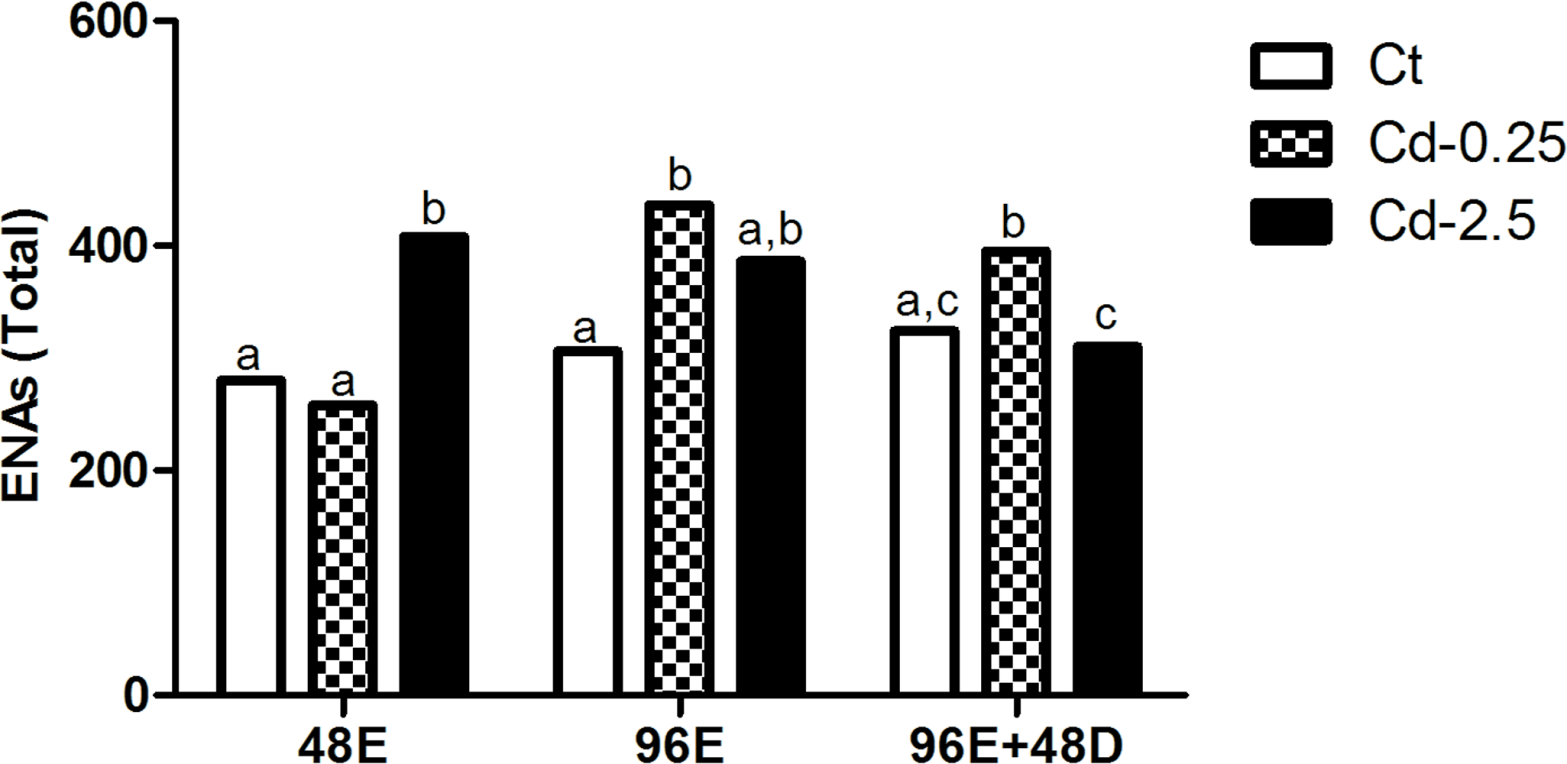
Total number of ENAs in *in vivo* assay. Values followed by different letters differ by Chi-square test (p < 0.05).

Considering all ENAs for each treatment, no difference was observed among the control groups; however, a significant increase of approximately 45% compared to control was observed at 48 hours of exposure to Cd-2.5 and a similar increase (43%) was observed at 96 hours of exposure to Cd-0.25. A significant decrease of 19.6% was seen only in the Cd-2.5 group between 96E and 96E+48D (Fig 6).

The increases in total ENAs showed a strong positive relationship with specific nuclear alterations (lobed, blebbed and vacuolated) that were time and concentration-dependent, whereas other nuclear alterations such as condensed and bud were negatively associated with the total ENA increase. Blebbed was associated with the increase of total ENAs in the Cd-0.25 group as the time of exposure increased (48E and 96E). However, the influence of vacuolated on ENAs at both exposure concentrations (Cd-0.25 and Cd-2.5) increased over 48 hours. Lobed had an influence during the depuration period (96E+48D). Condensed showed an inverse relationship with the total ENA frequencies during the entire exposure period; bud showed a similar pattern in the depuration period. Notched and micronuclei were not significantly related with the total ENA frequency in any treatment.

#### Cadmium content in total blood

**Fig 7:**
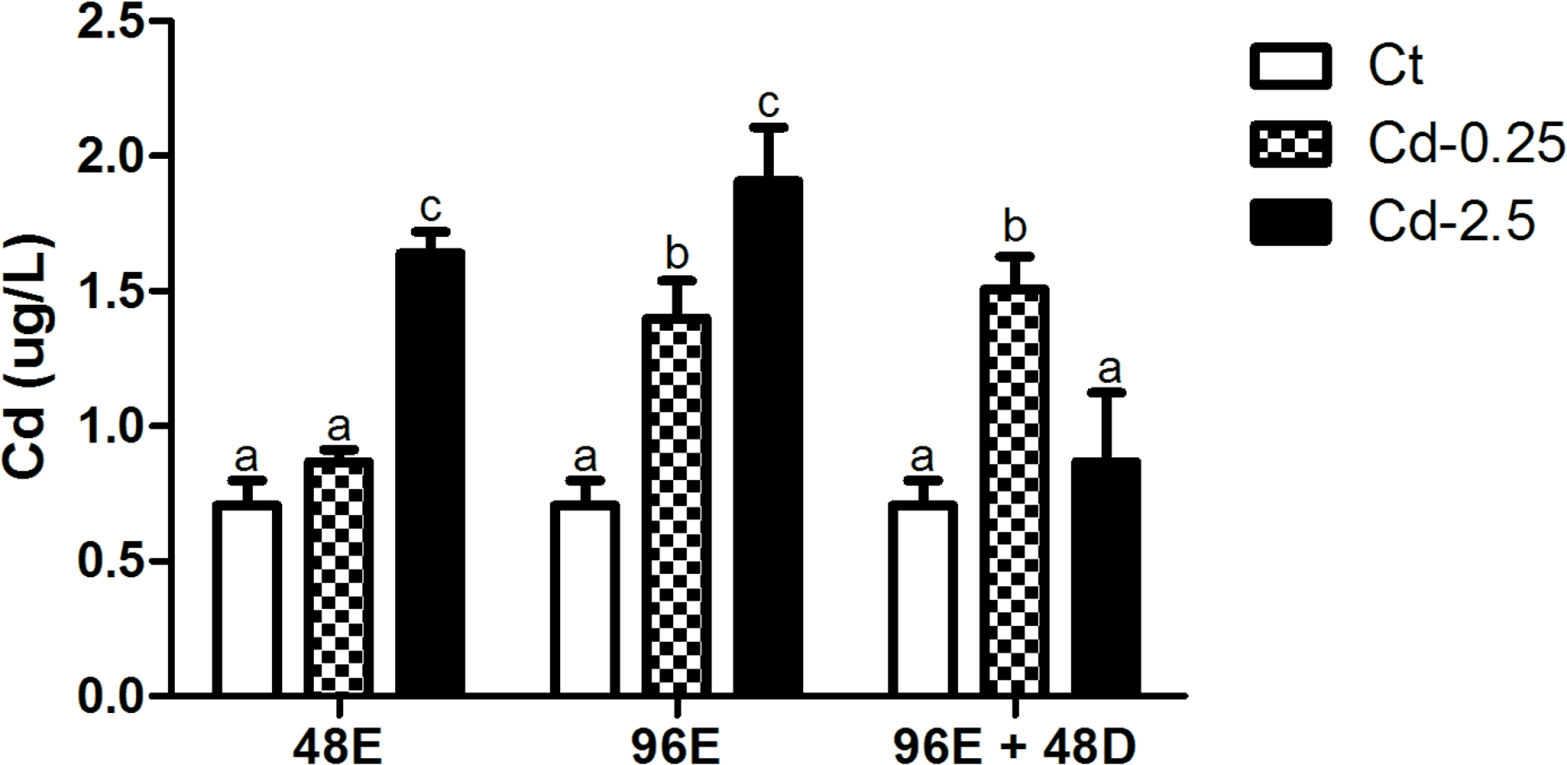
Concentration (means ± S.D.) of cadmium in blood obtained from animals of the *in vivo* assay. Values followed by different letters differ by ANOVA and Bonferroni tests (p<0.05).

No difference in blood Cd content was observed among controls during the assay.There was an increase of 2.3 times after 48h and 2.7 after 96h of exposure in the higher concentration treatment. These values were reduced to the control levels after depuration period. The increase of Cd content observed after exposure to 0.25 mg L^-1^ reach near 2 times the controls values at 96h and did not present depuration (Fig 7).

#### Light microscopy and morphometrical analysis in spleen and head kidney

**Fig 8:**
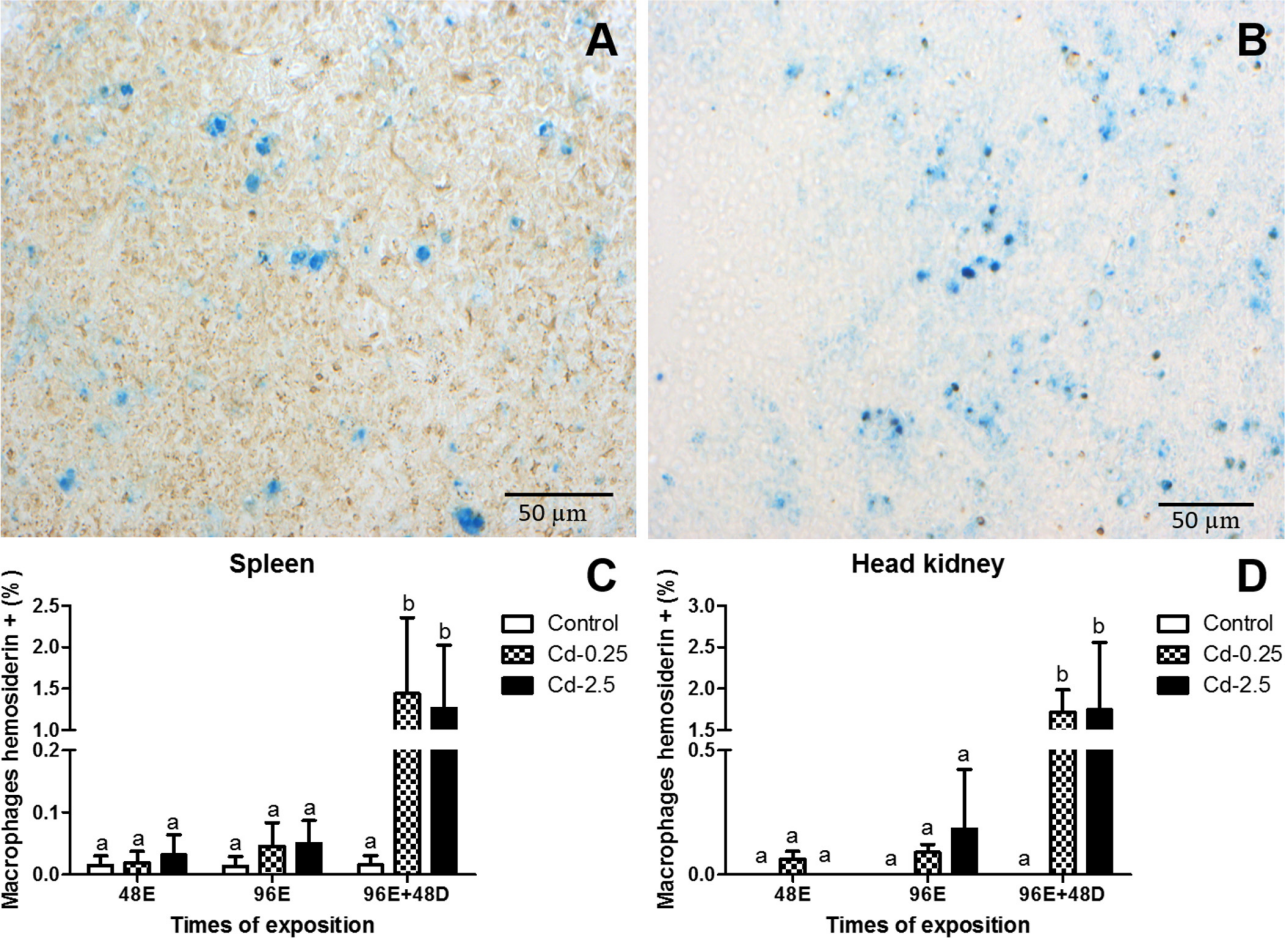
Single macrophages of spleen and head kidney of *O*. *niloticus* stained by Pearls’ histochemical technique. (A, B) Pigment-containing granules predominantly labeled with hemosiderin (blue). (C, D) Percentage of labeling for hemosiderin submitted to different treatments. Values followed by different letters differ by ANOVA and Bonferroni post test (p <0.05).

Through Pearl’s technique, it was possible to distinguish three types of labels: hemosiderin (blue), lipofuscin (yellow-brownish) and melanin (black). The hemosiderin is a pigment that show intracellular iron accumulation produced by the degradation of ferritin. This pigment was present in spleen and head kidney of single macrophages (Fig 8A and 8B) and in melanomacrophages centres.

In single macrophages it was possible to observe an increase in ferritin label and occurrence of the pigment in the spleen and head kidney only in depuration period (96E+48D) from fish exposed to Cd (Fig 8C and 8D).

**Fig 9:**
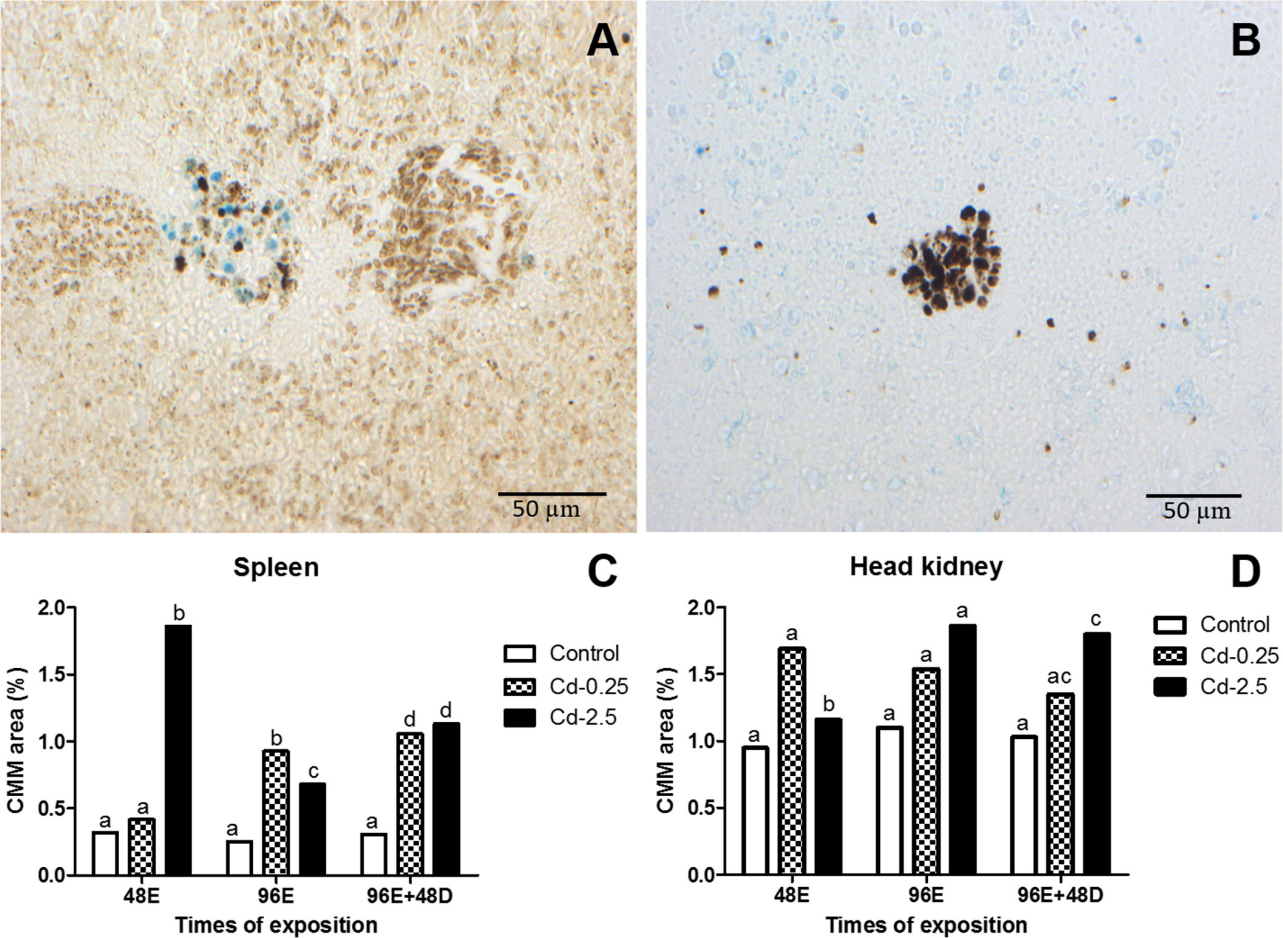
Melanomacrophage centres in spleen and head kidney of *O*. *niloticus* stained by Pearls’ histochemical technique. (A, B) MMC exhibiting lipofuscin (yellow-brownish) and melanin (black) pigments. (C, D) Percentage of the volumetric data melanomacrophage centres in tilapia submitted to different treatments. Values followed by different letters differ by Fisher test (p <0.05).

The occurrence of melanomacrophage centres (MMC) in spleen and head kidney (Fig 9A and 9B) shows an increase in the percentage areas of CMM after 48 hours of exposure in the higher concentration (Cd-2.5) with subsequent decrease in depuration period (Fig 9C and 9D). In spleen at lower concentration (Cd-0.25), this increase was seen after 96 hours and not in the depuration period. There was no difference between controls (Fig 9C and 9D).

#### Scanning electron microscopy and analytical microscopy (X-ray microanalysis)

**Fig 10:**
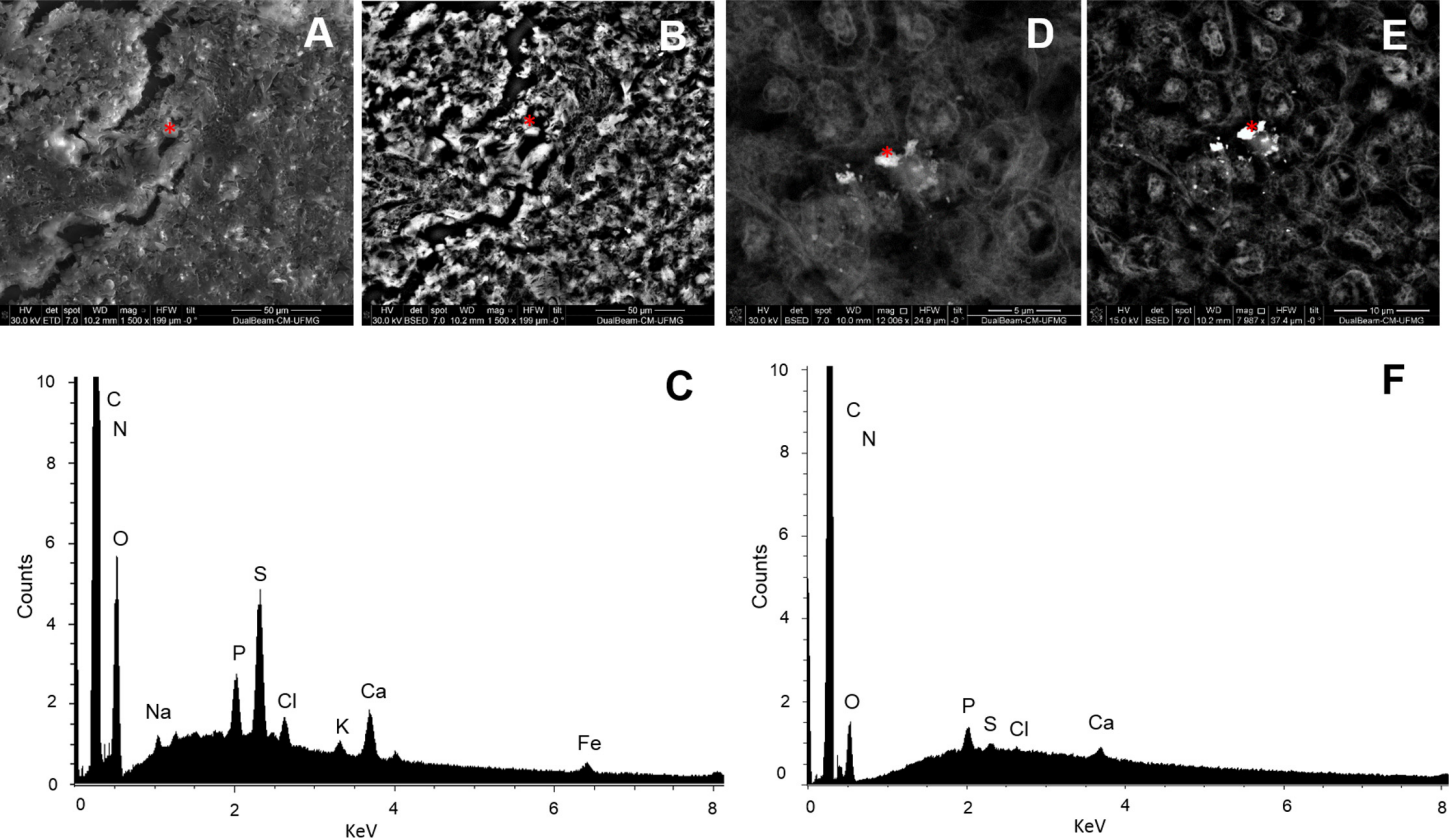
Spleen and head kidney MMCs. (A) Surface images obtained by secondary electrons from spleen and (D) head kidney tissue sections. The corresponding backscattered electron images of electron-dense granules from the red dots were identified in the respective images (B, E). The spectra obtained by X-ray microanalysis of the granule in spleen (C) and head kidney (F), showing characteristics peaks of C, O, P, S, Ca and Fe and C, O, P, S and Ca, respectively.

Scanning electron microscopy displaying the surface images of tissue sections acquired by secondary electrons in spleen (Fig 10A) and head kidney (Fig 10D). Electron-dense micro regions present inside cell tissue were determined through images with the backscattered electrons for spleens and head kidney (Fig 10B and 10E) respectively from animals exposed to cadmium.

The punctual x-ray microanalysis made in electron-dense micro regions of the spleen showed the spectra characteristic peaks of C, O, Na, P, S, Cl, K, Ca and Fe (Fig 10C). However, it was not possible to observe the presence of Fe in the spectra obtained from granules of head kidney (Fig 10F). The peaks observed in head kidney were C, N, O, P, S and Cl as exemplified by the control animal (Fig 10F).

## Discussion

Micronuclei and the other nuclear alterations can be considered effective pollution indicators. Some of their advantages are simplicity, reliability and sensitivity, therefore, they are considered as a powerful tool for the detection of genotoxic and cytotoxic damage [1–4, 51]. Despite these advantages in genotoxicity studies the ENAs relative frequencies are poorly accessed as genotoxic markers in time lapsed approach.

Their genesis have been associated to genotoxic agents and described as DNA double-strand breaks, displaying marked relationship between nuclear alterations and phosphorylation of ɤH2AX at serine 139 (c-H2AX) and nuclear lamin B1 [52]. According [53], these events are still associated with cytoplasmic membrane that pulls the chromatin out of the nucleus through the lamina break.

Considering that the teleosts erythrocytes present easy acquirement and the use of flow cytometry have been widely applied in recent years including in genotoxic studies [54], this present study showed tilapia erythrocytes position in flow cytometry graphs allowing their use with this technique.

Some species of fish, the position of blood cell populations have been determined in flow cytometry graphs [55–59]; however, no description of *O*. *niloticus* blood cells is available with this technique. These studies demonstrated that lymphocytes, thrombocytes, and granulocytes occupy a similar position in carp, zebrafish, and salmon. In these animals, red blood cells had the same size as thrombocytes and lymphocytes and were smaller than granulocytes. The size of *O*. *niloticus* blood cells was evaluated elsewhere [60] by Percoll gradient, enzymatic reactions, and immunocytochemistry studies. It was found that granulocytes and erythrocytes measured 9–13 μm, whereas lymphocytes and thrombocytes were 4.5–6 μm. This corroborates our other findings, and the flow cytometry analysis enabled the evaluation of our genotoxic assays.

This present study showed that erythrocytes from *O*. *niloticus* recover to basal levels the ROS and membrane permeability mismatching the ENAs that were closely associated to cadmium content. According to [15], cadmium induces oxidative stress by indirect processes, inducing decreases in cellular antioxidants and release of ROS by mitochondria. A reduction in cellular antioxidant levels could occur after exposure to Cd doses lower than 5 μM. Moreover, intoxication with < 50 μM Cd can lead to an early increase (15 minutes) in hydrogen peroxide levels, in turn affecting the permeability barriers of plasmatic membranes. In our study, erythrocytes in the Cd-0.25 and Cd-2.5 groups had more disrupted membranes over the first exposure interval, which was a faster response than that of the ENAs. [61] evaluated the effects of low pH (5.3) in *O*. *niloticus* blood cells and established a sequence of events of cellular damage on exposure to acidic environments: induction of oxidative stress through generation of ROS, lipid peroxidation by ROS action on fatty acids, and DNA damage that may be irreversible leading to cell death. Therefore, the specific ENA dynamics observed are quite distinct from those observed for membrane permeability biomarkers; rather, they are more related to the internal cadmium content in *O*. *niloticus*.

In addition, in the present study, the cadmium accumulation showed an intimate relationship with the occurrence of ENAs, which increased by up to *circa* two fold relative to the controls (1.40% in control and 2.85% in affected animals for ENAs and 0.70 to 1.91 μg·L^-1^ for blood cadmium content). This may indicate that the most susceptible cells are the erythrocytic precursor cells or erythrocytes in the final stages of maturation. It is therefore suggested that ENAs could be derived from the DNA replication process and maybe the main promoters of circulating ENAs. Cells with proliferative capacity have been shown to recurrently exhibit nuclear alterations [53, 61, 62, 63].

Few studies have shown that cadmium contamination is associated with increased frequency specific ENAs in fish erythrocytes. In *O*. *niloticus*, an increase in ENA frequency after the chronic exposure to 0.5 and 1.0 mg L^-1^ cadmium was previously described [64]. These increases were also observed in other species of fish with chronic exposure to cadmium at 0.1 mg L^-1^ [2] and 0.5 mg/kg, 1 mg/kg, and 2 mg/kg [65].

We also showed that ENAs displayed a specific response for each treatment, with a greater number of correlations at the highest exposure (see Table 5). As exposure times increased, blebbed was the nuclear alteration that influenced the increase of total ENAs. Additionally, vacuolated had the highest number of correlations with the treatments, which exerted an influence on the total ENAs as the exposure time increased. In addition, the frequency of blebbed and vacuolated increased over time at different Cd concentrations, suggesting that these nuclear alterations are influenced by exposure time and concentration.

When the animals were placed in depuration, the frequency of cells containing lobed nuclei decreased concurrently with the internal blood cadmium concentration. In addition, a positive correlation with blood cells stained by PI was observed. As consequence of their high frequency and the decrease in the total number of ENAs, it is possible that cells containing lobed presented compromised membrane permeability and were possibly removed from the bloodstream. Therefore, this nuclear alteration (lobed) appears only occur during the depuration period and not during the exposure period. In contrast, the cells with a condensed nucleus presented a faster decrease in frequency when the blood cadmium content increased, possibly because of their reduced life span. In contrast, bud was observed later than condensed and, similar to lobed, it can be considered a marker of depuration. In general, blebbed, vacuolated and lobed were the most important alterations during the exposure and depuration periods. In this study, nuclear alterations such as bud and condensed were also reliable markers for cadmium exposure, as they had an opposite response to the total ENA frequency and the cadmium content in the blood.

Two other nuclear alterations, micronuclei and notched, did not present responses to the exposure time and concentrations used in this study. Several studies [4, 51, 66–68] showed a significant increase of micronuclei in erythrocytes of animals that received different contaminants (e.g., mitomycin, cyclophosphamide, petroleum refinery effluents, pathogenic strains of fungi, and combined metals). In our study, micronuclei showed a very low incidence and no significant difference among treatments. A similar result was described by [65] and [22], in which the induction of micronuclei by heavy metal, including cadmium, in *Oncorhynchus mykiss* and *O*. *niloticus* was evaluated. In the study of [22], despite the lack of significant differences in rates of micronuclei in *O*. *niloticus*, increased rates of cells with breaks in DNA molecules were demonstrated by the Comet method. These data highlight the importance of the study of other ENAs.

The results presented herein show low concentrations of cadmium in the blood (mM) and are unique in that they determine the concentration of Cd in the total blood of fish, particularly *O*. *niloticus*. In fish, studies focusing on cadmium content are frequently performed in the liver, kidney, gill and muscle [69–72]. In this study, blood cells appeared to have a temporary accumulation of cadmium with differences in concentration and dynamics compared to that of other organs such as the kidney, liver, spleen and muscle. [71, 73]. In general, studies performed to determine the content of cadmium in fish have shown a relationship between Cd and cellular alterations such as decreased cellular viability, increased membrane permeability, apoptosis induction, increased ROS, induction of breaks in DNA strands, increased expression of repair genes and the oxidative stress defense [69, 74, 75].

Moreover, the decrease on ENAs frequencies on blood stream after removal of the animals from cadmium exposure could be associated to cell cycle arrest impairment in the hemocytopoetic organ. Studies have demonstrated that in the range of cadmium content observed in this study, cadmium-induced cell death (apoptosis) is associated with increased p53 protein and p53 mRNA levels in different cell lines [15, 76]. According to [77], the caspase-dependent apoptotic pathway appears to be associated with lower doses (5 μM) of cadmium with short exposure; however, doses of cadmium higher than 50 μM could lead to necrosis. Studies on kidney cells have described that these cells respond to DNA damage by stopping the cell cycle to facilitate repair of DNA or by signaling apoptosis to remove damaged cells. Thus, the repair systems are activated and specific enzymes reverse the damage or the apoptotic process is initiated. However, cadmium appears to disrupt the conformation of p53, and cells exposed to cadmium can either enter mitosis or initiate replication of DNA, causing genetic instability [76]. The events described above could be factors that act directly on the frequency of some ENAs, such as condensed and lobed.

Studies of melanomacrophages have identified cells containing phagocytosed red blood cells in spleen [78] and hematopoietic cells in anterior kidney [8, 9]. The structure and cellular content of MMCs and single macrophages in kidney and spleen of *O*. *niloticus* was described and compared. We suggest that the kidney and spleen single macrophages are directly involved in the regular storage, relocation and recycling of iron-compounds of damaged red blood cells, whereas the MMCs present functional dissimilarity in spleen and head kidney. In fact, [79] suggested that cells labeled by hemosiderin are able to play a role in cleansing the circulation of damaged red blood cells by storage of iron-compounds. This hypothesis was reinforced with the increase of hemosiderin pigment specifically from hemocateretic macrophage activity. The two organs studied were very similar in hemosiderin percentage labeling and inversely related to the results of ENAs frequencies in depuration period. Some others studies corroborated these findings, associating the presence of hemosiderin in different organs of teleosts with phagocytic events in single macrophages [80] and MMCs [7, 81].

The morphological appearance, size and cellular contents of MMCs in teleosts have suggested that MMC can increase under stress conditions [82–86]. According to our results, the MMC area increase in spleen and head kidney during the cadmium exposure but does not relate with the hemosiderin content. The area increase in MMCs in spleen occurred in early time like membrane disruption and ROS. The hemosiderin observed in these MMCs indicates that their involvement in depuration of damaged red blood cells. This function also is shared by single macrophages. In head kidney, the MMCs display different behavior and showed a progressive increase. These results suggest an important functional difference between MMCs in these two organs thus demonstrating the important role of spleen MMCs in iron metabolism.

These findings suggests the time lapsed vision about the main blood cell component (erythrocytes) and their systemic behavior involving hemocytopoetic and hemocateretic aspects after their interactions with different concentrations of cadmium as genotoxic agent.

This study showed that cadmium concentration affects directly the cell cycle, as well as, the macrophages iron turn over in MMC or in single macrophages mainly in spleen parenchyma, affecting the ENAs frequencies differentially.

## Conclusions

Cd content in the blood of *O*. *niloticus* changes the membrane permeability and ENA frequencies in different manners. The detoxification effects of these cellular alterations occur in this fish when it is removed from the contaminant, thus leading to a decrease in cells with membrane disruption and altered nuclei, accompanied by cadmium blood content. Each ENA showed a specific dynamic and correlation, enabling a better understanding of the pattern of behavior and indicating the probable fate of cells containing this type of nuclear alteration. The data obtained in this study shed light on a possible mechanism underlying the occurrence of different ENAs in genotoxicity studies. The present study enabled the evaluation of the close relationship of ENAs with the cadmium blood content and the time-related mismatch with membrane permeability changes and recovery.
